# Prevalence and spatial distribution characteristics of human brucellosis in Ningxia from 2010 to 2024

**DOI:** 10.1371/journal.pntd.0013910

**Published:** 2026-02-03

**Authors:** Hongju Duan, Xianglin Wu, Zhenhua Lei, Yu Zhao, Tianbo Ma, Tinglong Yang, Zhiyi Wang, Mingzhe Jiang, Liying Wang, Xueping Ma

**Affiliations:** 1 Department of Parasitic Diseases and Brucellosis Control, Ningxia Hui Autonomous Region Center for Diseases Control and Prevention, Yinchuan, Ningxia, China; 2 School of Public Health, Ningxia Medical University, Yinchuan, Ningxia, China; 3 NHC Key Laboratory of Metabolic Cardiovascular Diseases Research, Yinchuan, Ningxia, China; 4 Ningxia Key Laboratory of Environmental Factors and Chronic Disease Control, Yinchuan, Ningxia, China; 5 National Institute of Parasitic Diseases, Chinese Center for Disease Control and Prevention; Chinese Center for Tropical Diseases Research, Shanghai, China; 6 National Key Laboratory of Intelligent Tracking and Forecasting for Infectious Diseases, Shanghai, China; 7 Key Laboratory on Parasite and Vector Biology, Ministry of Health, Shanghai, China; 8 WHO Collaborating Centre for Tropical Diseases, Shanghai, China; 9 National Center for International Research on Tropical Diseases, Ministry of Science and Technology, Shanghai, China; Colorado State University, UNITED STATES OF AMERICA

## Abstract

**Background:**

Brucellosis, a neglected zoonotic disease as defined by the World Health Organization (WHO), represents a substantial burden, causing both severe human morbidity and significant economic losses to the livestock industry. In recent years, the Ningxia Hui Autonomous Region (Ningxia) in northwest China has emerged as a region with persistently high endemicity of human brucellosis. An in-depth analysis of the epidemic’s prevalence and spatiotemporal evolution in this region is essential to inform the optimization of targeted prevention and control measures.

**Methods:**

Data on all human brucellosis cases from 2010 to 2024 were collected from the China Information System for Disease Control and Prevention. Temporal trends, global and local spatial autocorrelation, and spatial scan statistics were used to explore temporal patterns, regional clustering, and high-risk areas.

**Results:**

A total of 35 665 human brucellosis cases were reported in Ningxia, with no associated deaths, during the study period. The average annual incidence rates was 35.08/100,000, ranging from 3.31 to 84.80 per 100,000. Two incidence peaks were observed: the first in 2015 (43.66/100,000) and the second in 2022 (84.80/100,000). The human brucellosis incidence rates showed that overall increasing trend from 2010 to 2024 (average annual percentage change [AAPC]=18.31%, but this was not statistically significant (t = 1.63, *P* = 0.102). Male accounted for 71.01% (25 325 cases) of all cases, 2.45 times higher than females (28.99%, 10 340 cases). Cases were reported in all 22 counties of Ningxia. The top five counties with the highest average annual incidence were: Yanchi County (184.79/100,000), Tongxin County (75.91/100,000), Zhongning County (46.47/100,000), Xiji County (46.15/100,000) and Yuanzhou District (45.30/100,000), while Xingqing District had the lowest rate (5.60/100,000). The top five counties with the fastest AAPC were: Huinong District (AAPC = 140.46%), Qingtongxia City (AAPC = 127.19%), Pengyang County (AAPC = 111.19%), Haiyuan County (AAPC = 107.97%) and Lingwu County (AAPC = 102.04%). Global spatial autocorrelation analysis revealed spatial clustering of human brucellosis incidence between 2022–2024 (Moran’s *I* 0.266, p = 0.033; 0.394, p < 0.001; 0.353,p = 0.002, respectively). Local spatial autocorrelation identified “high-high” clusters primarily in Zhongning County, Tongxin County and Lingwu County. Additionally, spatial scanning analysis detected four spatial clusters, including one most likely cluster (LLR = 6474.66, RR = 3.71, *P*  ( 0.001) and three secondary clusters. The primary cluster center was located at Jingyuan County (38.10°N, 106.34°E), with a cluster radius of 271.44 km.

**Conclusion:**

Our findings reveal a substantial burden of human brucellosis in Ningxia. The disease exhibits distinct seasonality, peaking in summer and autumn. Priority interventions should include: (1) Enhanced health education and targeted behavioral interventions for middle-aged and elderly males to improve personal awareness and competency in prevention and control; and (2) Timely identification of key risk factors and implementation of tailored prevention strategies in regions experiencing either a rapid increase or persistently high incidence.

## Author summary

Brucellosis represents a substantial burden, causing both severe human morbidity and significant economic losses to the livestock industry. From 2014 to 2021, Ningxia had the second-highest incidence rate of human brucellosis in China, and it rosed to the top in 2022. Clarifying the epidemic characteristics and spatiotemporal characteristics of brucellosis is crucial for targeted intervention. We analysed 15 years (2010–2024) of surveillance data, covering 22 counties and 35,665 confirmed cases.Joinpoint regression was used to clearly identify disease trends.Spatial autocorrelation (global/local Moran’s I) and spatiotemporal scan statistics (SaTScan) were applied to detect clustering patterns. The human brucellosis incidence rates showed that overall increasing trend from 2010 to 2024 (average annual percentage change [AAPC]=18.31%. Cases were reported in all 22 counties of Ningxia. Yanchi County has the highest average annual incidence rate.There were five regions where AAPC exceeds 100%.Global spatial autocorrelation analysis revealed spatial clustering of human brucellosis incidence between 2022–2024. Local spatial autocorrelation identified “high-high” clusters primarily in Zhongning County, Tongxin County and Lingwu County. Additionally, spatial scanning analysis detected four spatial clusters and three secondary clusters. For the first time, the overall trend of brucellosis incidence in Ningxia has been clearly defined, and areas with rapid incidence and key regions have been identified.We recommend precision strategies:enhanced health education,targeted behavioral interventions,identification of key risk factors and implementation of tailored prevention strategies.

## Introduction

Brucellosis is a zoonotic disease caused by a Gram-negative [1,2], facultative intracellular bacteria of the *genus Brucella* [3]. Transmission typically occurs through direct contact with infected animals and their secretions [4], or via consumption of unpasteurized dairy products [2]. Clinical manifestations in humans commonly include fever, sweating, fatigue, and arthritis [5,6]. Individuals residing in or traveling to endemic regions constitute the primary high-risk populations [7,8]. Since its initial isolation from *Brucella melitensis* by British bacteriologist David Bruce in 1887 [9], brucellosis has become endemic worldwide. Current epidemiological data indicate its presence in over 170 countries and territories [10,11]. Global annual incidence exceeds 500,000 reported cases; however, the true disease burden may be 10–25 times higher due to underdiagnosis and inadequate surveillance systems [12]. Beyond its significant health impact [13], brucellosis causes substantial economic losses in the livestock industry, estimated at billions of US dollars annually [14], representing a major global public health challenge [6]. Multiple factors influence the transmission dynamics of brucellosis. Meteorological parameters affect disease occurrence through their impact on: (1) agricultural practices, (2) bacterial survival in the environment, and (3) vector ecology [15]. Recent studies demonstrate significant positive correlations between wind velocity and brucellosis incidence, while revealing inverse relationships with mean temperature and relative humidity [14,16]. Furthermore, various socioeconomic and environmental determinants - including the Normalized Difference Vegetation Index (NDVI), Gross Domestic Product (GDP) per capita, and livestock (particularly ovine) population density - have been identified as key modulators of disease transmission [17,18].

Since 1995, China has experienced a significant resurgence of brucellosis, characterized by a sustained upward trend in reported cases and progressive geographical expansion to all provinces (including municipalities and autonomous regions) [19]. Epidemiological studies have demonstrated substantial spatiotemporal heterogeneity in human brucellosis transmission patterns [20]. During 2012–2019, the epidemic was predominantly concentrated in northern China [21], especially in Inner Mongolia and surrounding provinces/autonomous regions [22,23].

Ningxia has witnessed particularly rapid disease proliferation since reporting its first human case in 2004. The reported incidence rate surged dramatically from 0.02 cases per 100,000 population in 2004 to 44.84 cases per 100,000 in 2015 - representing a > 2,000-fold increase. By 2014, all 22 counties (including cities and districts) in the region had become high-prevalence zones, ranking second nationally in disease burden [24]. Following implementation of large-scale livestock immunization programs (2016–2018) under the National Brucellosis Prevention and Control Plan by the Ningxia Department of Agriculture and Rural Affairs, human cases temporarily declined. However, resurgence occurred subsequently, with Ningxia ranking first nationally in brucellosis incidence for consecutive years in 2022 and 2023. To address this severe epidemic, the Ningxia government enacted the “Special Three-Year Action Plan for Brucellosis Prevention and Control (2022-2024)”. While these measures have achieved some containment, current prevention strategies remain predominantly generalized [25], lacking: (1) precise identification of high-risk populations, (2) geographical targeting of transmission hotspots, and (3) differentiated intervention approaches. This non-specific resource allocation paradigm results in suboptimal utilization of public health resources, diminished intervention efficiency, and challenges in achieving sustainable epidemic control.

Consequently, precise characterization of the epidemiological features, long-term trends, and spatiotemporal dynamics of human brucellosis in Ningxia is crucial for optimizing intervention strategies and enabling targeted resource allocation. This study utilized regional surveillance data (2010–2024) to conduct comprehensive analyses through: (1) temporal trend analysis, (2) spatial autocorrelation examination, and (3) spatiotemporal scan statistics. These methodologies were employed to systematically investigate the epidemic’s epidemiological characteristics, evolutionary patterns, and spatial clustering phenomena. The findings provide an evidence-based foundation for implementing precision prevention measures and optimizing public health resource deployment in Ningxia and other endemic regions with comparable epidemiological profiles.

## Methods

### Ethics approval and consent to participate

This study has received ethical approval from the Ningxia Center for Disease Control and Prevention Ethics Committee with IRB the number 2025-LLSC-190.

### Study area

Ningxia is one of China’s five provincial-level autonomous regions, is situated in the upper reaches of the Yellow River in northwestern China (38°25’N-41°38’N, 104°17’E-107°39’E). The region administratively comprises 5 prefecture-level cities and 22 counties (including county-level cities and districts). It shares borders with Shaanxi Province to the east, Inner Mongolia Autonomous Region to the west and north, and Gansu Province to the south, covering a total area of 66,400 km^2^. As of 2023, the registered population reached 7.28 million inhabitants. The region has a temperate continental semi-arid climate.

### Source of data

Human brucellosis case data in Ningxia from January 1,2010 to December 31,2024 were obtained from the “China Disease Control and Prevention Information System Infectious Disease Surve System”. Established in 2004, this system covers medical and health institutions at and above the township level nationwide. According to the Law on the Prevention and Control of Infectious Diseases, all medical and health institutions across the country must report case information, including age, gender, occupation, current residential address, date of symptom onset, within 24 hours of meeting the diagnostic criteria for brucellosis in China (WS 269-2019). All reported cases are reviewed by the Centers for Disease Control and Prevention at fourth administrative levels: county, city, provincial, and national. County-level electronic maps of Ningxia were downloaded from the National Geographic Information Public Service Platform.

### Statistical analysis

#### Joinpoint regression analysis.

A Joinpoint regression model was specifically designed to analyze trend changes in time series data [26]. By finding one or more “connection points” in the data, the entire study period is divided into multiple sub-intervals, within which the data trend is relatively consistent, allowing for a more detailed analysis of temporal changes. The Joinpoint regression model was constructed with the natural logarithm of the reported incidence as the dependent variable and the year as the independent variable. The number and location of turning points were determined based on the grid search method (GSM), and the Monte Carlo permutation test and modified Bayesian information criterion (MBIC) were used. The model with the smallest MBIC value was selected as the optimal model. The average annual percent change (AAPC), average percent change (APC) and their 95% confidence interval (CI) were calculated to evaluate the trend in brucellosis incidence and determine statistical significance [25,27]. AAPC is used to comprehensively evaluate the overall mean trend of incidence over a period of time, and APC is used to describe the trend of incidence over a period of time. When the number of turning points is 0, AAPC = APC. If AAPC >0 or APC > 0, it indicates that the disease is on the rise during that time period, if <0 is on a downward trend, = 0 means that there is no obvious trend [28].

#### Spatial autocorrelation analysis.

Spatial autocorrelation analysis is used to quantify the aggregation characteristics of spatial element attribute values and reveal the direction and intensity of spatial correlation of variables. It can be divided into Global Spatial Autocorrelation and Local Spatial Autocorrelation [29]. Global spatial autocorrelation is evaluated by calculating the Moran’s *I*, and its value range is [-1, 1], which characterizes the correlation strength, positive values indicate high-high or low-low homogeneity aggregation, negative values reflect high-low or low-high heterogeneity discreteness, and =0 conforms to the random distribution hypothesis [30]. However, global correlation analysis ignores the existence of spatial heterogeneity and can only measure the overall correlation, but cannot indicate specific aggregation areas. The local spatial autocorrelation (LISA) was used to identify the unit-level spatial association model, and four types of heterogeneity were divided: high-high (HH) and low-low (LL) represented homogeneous aggregation, and high-low (HL) and low-high (LH) reflected heterogeneous discreteness [31,32]. In this study, the global spatial autocorrelation analysis was carried out first, and then the local spatial autocorrelation analysis was performed.Global and local spatial autocorrelation Moran’s I values were calculated and visualized using tools within the Spatial Statistics Toolbox of ArcGIS version 10.3 and using a local spatial association metric cluster map.

#### Spatial scan clustering.

It is a data analysis method based on mobile mapping window, which has been widely used to detect spatiotemporal clusters of diseases. Taking the county (district) as the geographical unit and the year as the time unit, the test statistics were constructed based on the Poisson distribution discrete scanning statistical model, and the empirical P value (*P* < 0.05) was calculated by Monte Carlo simulation (999 random sampling) to determine the statistically significant aggregation area. The maximum spatial scanning area was set at 50% of the total population and the maximum scanning time was set at 50% of the total duration of the study. The theoretical incidence was estimated based on the actual number of cases and the actual population, and the logarithmic likelihood ratio (LLR) was constructed based on the actual and theoretical cases inside and outside the scanning window. The window with the highest LLR value is defined as the most likely cluster. Other clusters that show statistically significant LLR are defined as secondary clusters. The relative risk of each agglomeration area was further calculated(relative risk, RR) [33].When test hypothesis *P* < 0.05 of the log likelihood ratio (LLR) was considered to indicate spatiotemporal clustering, the main clustering area was the one with the largest LLR value, while other statistically significant areas were secondary clustering areas [34].

### Statistical analysis

Use Excel software to verify and clean the collected case data. SPSS 21.0 (IBM, Armonk, USA) was used for descriptive epidemiological analysis, chi-square test was used for comparison of count data. R software was used to break down the time series of the number of cases in the past years.The incidence trend was using the Joinpoint 5.0.2 software (version 4.9.1.0; National Cancer Institute, Rockville, MD, USA).Spatial autocorrelation analysis was performed using the Arcgis 10.3 (Esri Inc., Redlands, CA, USA), software. Spatiotemporal scanning analysis was conducted using SaTScan software (v10.1.2, Boston, Massachusetts, USA).

## Results

### Epidemiological characteristics

**Fig 1:**
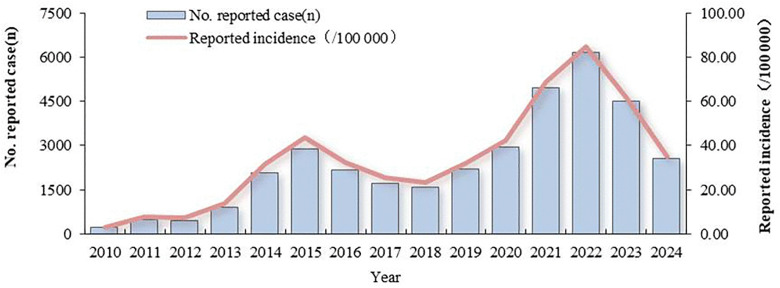
The incidence of human brucellosis in Ningxia from 2010 to 2024.

From 2010 to 2024, a total of 35 665 human brucellosis cases were reported, with no associated deaths. The average annual incidence was 35.08/100,000, ranging from 3.31 to 84.80 per 100,000. The AAPC was18.31% during 2010–2024,but it was not statistically significant (t = 1.63, *P* = 0.102).Two incidence peaks were observed: the first in 2015 (43.66/100,000) and the second in 2022 (84.80/100,000) (Fig 1).

**Fig 2:**
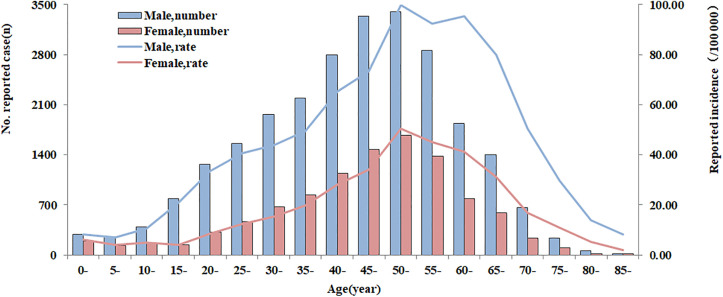
The incidence of human brucellosis in Ningxia from 2010 to 2024.

Incidence varied between years significantly (χ^2^ = 15126.25, *P* < 0.001). Among the reported cases, 25 325 males (71.01%) and 10 340 females (28.99%), yielding a male-to-female ratio of 2.45:1. Age-stratified analysis demonstrated brucellosis occurrence across all age groups, with particularly elevated incidence rates observed in the 35–74 year age bracket. The incidence rate of males was higher than that of females in all age groups (χ^2^ = 49.55, *P* < 0.001), and the average annual incidence rate of males (48.81/100,000) was 2.35 times that of females (20.77/100,000) (Fig 2).

**Fig 3:**
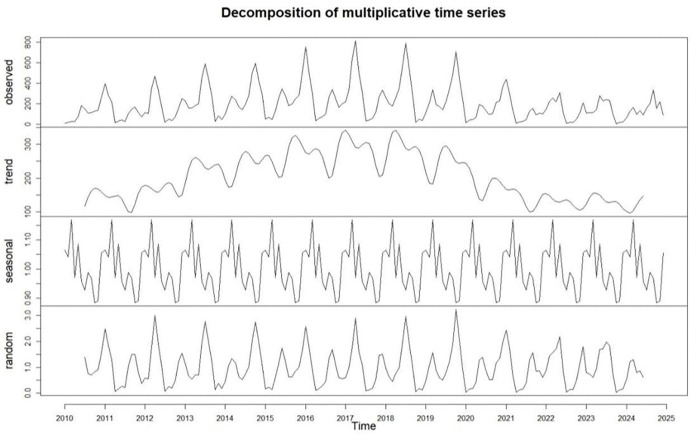
Seasonal and long-term trends of human brucellosis in Ningxia from 2010 to 2024. (Please note: The ‘trend’ and ‘random’ components at the sequence endpoints are shown as truncated because reliable estimates cannot be made. The ‘observed’ and ‘seasonal’ components display the data for the entire period.).

The monthly number of cases of brucellosis from 2010 to 2024 was decomposed, and the results showed an initial upward trend followed by a period of short-term stability and then a decline. At the same time, the disease exhibits distinct seasonality. From February to September, the number of reported cases was highest, accounting for 78.03% (27 829/35 665) of the total, with the fewest cases reported in December and peak in June (Fig 3).

**Fig 4:**
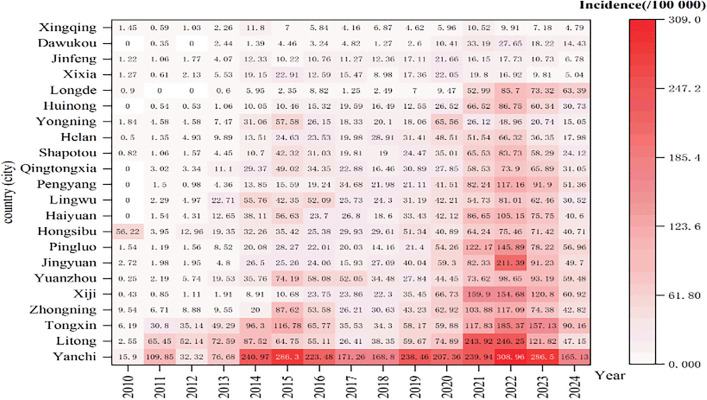
Incidence heatmap of human brucellosis in 22 regions in Ningxia from 2010 to 2024.

**Fig 5:**
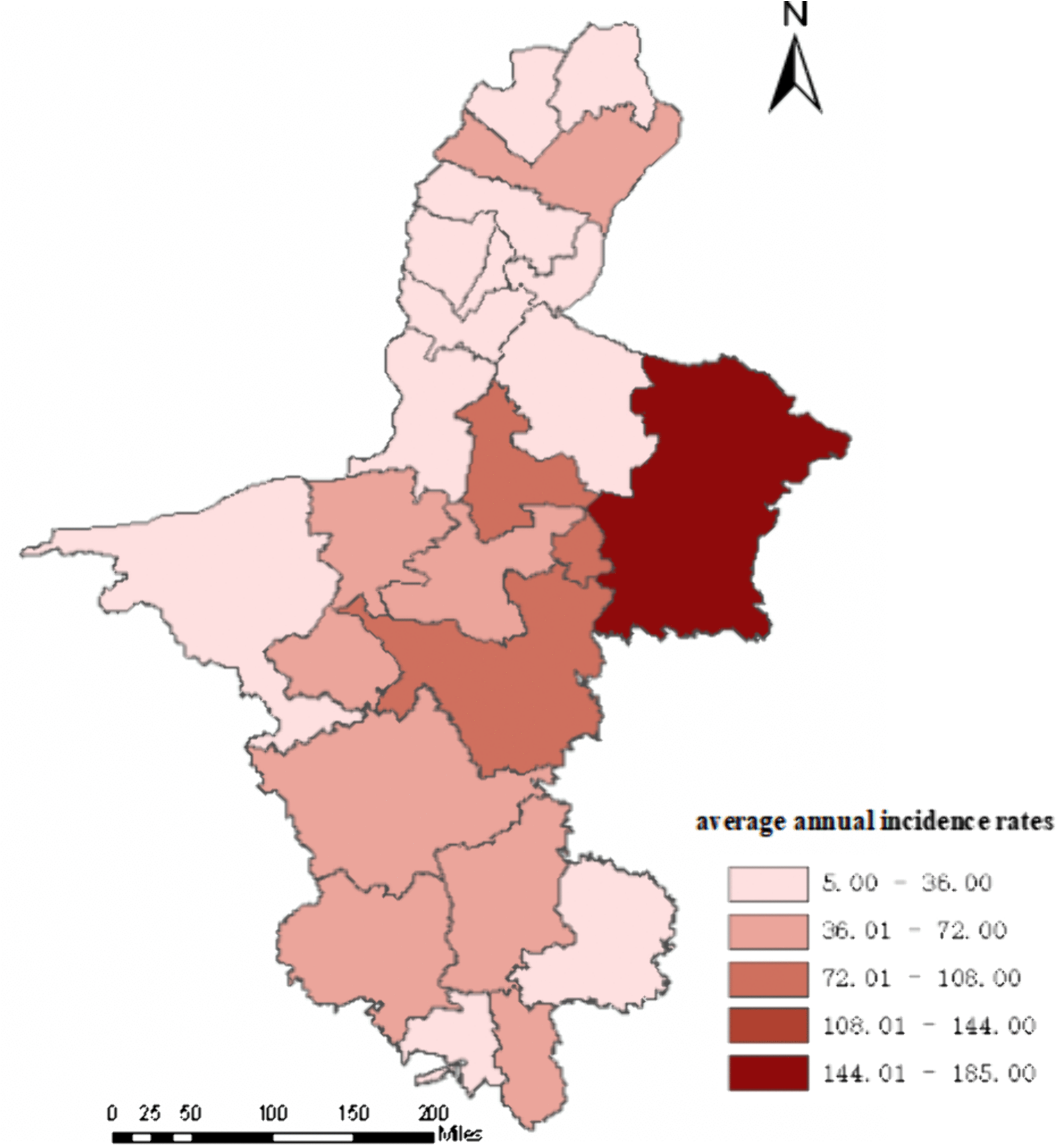
Average annual incidence rates of human brucellosis in 22 regions in Ningxia from 2010 to 2024 (https://www.webmap.cn/main.do?method=index).

**Fig 6:**
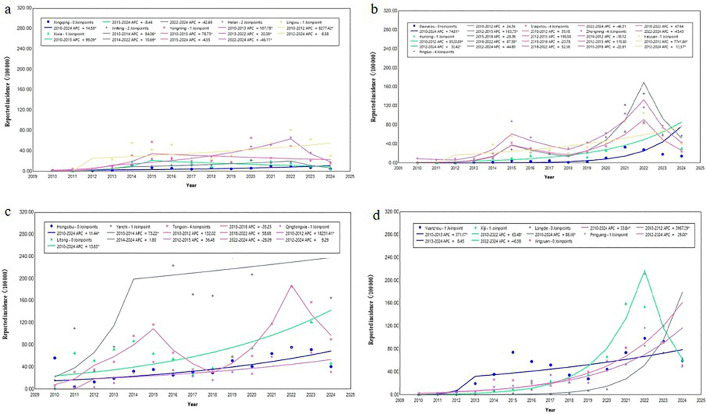
Trend of human brucellosis cases in 22 regions of Ningxia from 2010 to 2024.

All 22 counties reported cases (Fig 4). The five counties with the highest average annual incidence rates were Yanchi County (184.79/100,000), Tongxin County (75.91/100,000), Zhongning County (46.47/100,000), Xiji County (46.15/100,000), and Yuanzhou District (45.30/100,000); Xingqing District had the lowest rate (5.60/100,000) (Fig 5). The five counties with the fastest average annual growth trends were Huinong District (AAPC = 140.46%, t = 5.91, *p* < 0.001), Qingtongxia City (AAPC = 127.19%, t = 7.90, *p* < 0.001), Pengyang County (AAPC = 111.19%, t = 7.64, *p* < 0.001), Haiyuan County (AAPC = 107.97%, t = 6.61, *p* < 0.001), and Lingwu County (AAPC = 102.04%, t = 8.02, *p* < 0.001) (Fig 6).

### Spatial distribution of human brucellosis in Ningxia

As shown in Table 1, the global spatial autocorrelation analysis revealed a dynamic pattern of clustering. While statistically significant clustering (p < 0.05) was consistently observed from 2022 to 2024, several earlier years (notably 2010, 2013, 2014, 2019) exhibited p-values very close to the significance threshold (p < 0.10). This pattern suggests a underlying weak spatial dependency that intensified and became unequivocally significant in the most recent years of the study period (Table 1). The results of Local Indicators of Spatial Association (LISA) clustered distribution map demonstrated that in 2010, 2011, and 2013, there were 2, 1 and 1 counties, respectively, classified as “high-high” clusters. These cluster were located in Zhongning County, Tongxin County and Lingwu County. No “high-high” clusters were observed after 2013. The “low-low” clusters were mainly distributed in the northern and southern region of the Ningxia before 2016. Overtime, the “low-low” clusters in the north gradually shifted southw downward over time, while the “low-low” clusters in the southern disappeared.

**Table 1:** Results of the spatial autocorrelation test on human brucellosis cases in Ningxia from 2010 to 2024.

Year	Moran^,^s I	Z score	*P* value	clustered
2010	0.112	1.783	0.075	No
2011	0.041	0.747	0.455	No
2012	0.148	1.441	0.150	No
2013	0.188	1.690	0.091	No
2014	0.142	1.707	0.088	No
2015	0.106	1.354	0.176	No
2016	0.139	1.847	0.065	No
2017	0.102	1.626	0.104	No
2018	0.071	1.339	0.181	No
2019	0.110	1.710	0.087	No
2020	-0.009	0.342	0.732	No
2021	0.200	1.716	0.086	No
2022	0.266	2.135	0.033	Yes
2023	0.394	3.344	0.001	Yes
2024	0.353	3.063	0.002	Yes

### Identification of clusters for human brucellosis in Ningxia

**Fig 7:**
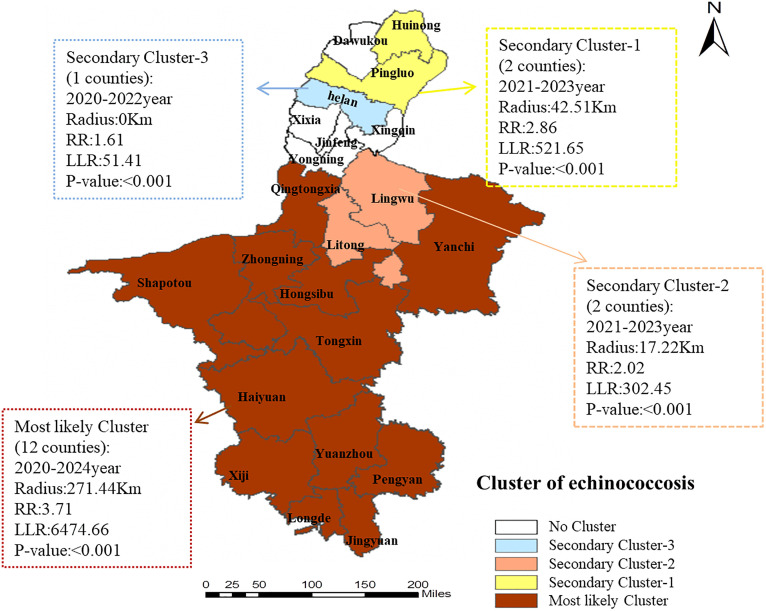
Results of the spatial autocorrelation test on human brucellosis cases in Ningxia from 2010 to 2024 (https://www.webmap.cn/main.do?method=index).

Spatiotemporal cluster analysis revealed that four high-risk clusters were identified across 22 counties (cities/districts) in Ningxia from 2010 to 2024. These clusters included one most likely cluster and three secondary clusters, involving a total of 17 counties (cities/districts). The most likely cluster was located in the central and southern of Ningxia, covering 12 counties (districts): Yanchi County, Tongxin County, Yuanzhou District, Xiji County, Zhongning County, Haiyuan County, Sha po tou District, Hong si bu District, Qingtongxia District, Pengyang County, Jingyuan County and Longde County. The cluster center was located at Jingyuan County (38.10°N, 106.34°E), with a cluster radius of 271.44 km. The cluster period was from 2020—2024 (LLR = 6474.66, RR = 3.71, P ＜ 0.001).The secondary cluster-1 was located at Hui nong District and Pingluo County with a radius of 42.51 kilometers (Table 2 and Fig 7).

**Table 2:** Spatial-temporal scanning analysis results of human brucellosis at the county level in Ningxia from 2010 to 2024.

Cluster	Time frame	Cluster center/radius(km)	Population	No.Cluster areas	Number of cases	Expected cases	*RR*	*LLR*	*P*
Most likely cluster	2020-2024	(35.50N, 106.33E)/ 271.44 km	3270831	12 (Yanchi County, Tongxin County, Yuanzhou District, Xiji County, Zhongning County, Haiyuan County, Sha po tou District, Hong si bu District, Qingtongxia District, Pengyang County, Jingyuan County and Longde County)	14583	5600	3.71	6474.66	<0.001
Secondary cluster	2021-2023	(39.24N, 106.78E)/ 42.51 km	461422	2 (Huinong District, Pingluo County)	1327	475	2.86	521.65	<0.001
2nd Secondary cluster	2021-2023	(38.102690N, 106.340050E)/ 17.22 km	704223	2 (Lingwu City, Litong District)	1573	797	2.02	302.45	<0.001
3nd Secondary cluster	2020-2022	(38.554430N, 106.349850 E)/ 0 km	270911	1 (Helan County)	530	330	1.61	51.41	<0.001

## Discussion

Human brucellosis is a serious public health problem in Ningxia. The study analyzed the flow characteristics, epidemic trends and spatiotemporal clustering of brucellosis. The results showed that the average annual incidence rate of human brucellosis disease in Ningxia was 35.08/100,000, which was significantly higher than the national average [23], Inner Mongolia [35], Shandong [36] and other regions [37–39].

From 2010 to 2024, the AAPC was18.31%, while the raw numbers clearly show an increase, the statistical test suggests this trend could be due to chance when considering the model’s fit. Meanwhile there were two peaks in 2015 (43.66/100,000) and 2022 (84.80/100,000). This trend is generally consistent with national data [40], and that from Xinjiang [41]. The 2015 peak may be related to the large-scale expansion of animal husbandry during Ningxia’s 12th Five-Year Plan period. According to the Ningxia Statistical Yearbook, between 2010–2024, the average annual growth rate of cattle and sheep population was 4.4%, and the average annual growth rate of beef and sheep meat production was 12.2%. From 2015 to 2018, the incidence of human brucellosis in Ningxia decreased, mainly because China issued the “National Brucellosis Prevention and Control Plan” in 2015 and the implementation of compulsory immunization of live vaccine against S_2_ strain of brucellosis for all sheep (except breeding sheep) by the Ningxia Department of Agriculture and Rural Affairs. These measures effectively prevented and controlled the spread of human brucellosis in sheep, and disrupted the “sheep-to-human” transmission chain, leading to a sharp decline in human cases. However, after 2019, the mass immunization was not continued. With the rapid development of animal husbandry, the number of cattle and sheep has increased sharply, while the public awareness and protective behavior did not improve accordingly. Consequently, the epidemic rebounded, and the incidence reached a historical peak (84.80/100,000) in 2022. After 2022, with the implementation of prevention and control plans such as the “Ningxia Brucellosis Prevention and Control Special Three-Year Action Implementation Plan (2022—2024)” [21] and the “National Plan for the Prevention and Control of Zoonotic Diseases among Animals (2022—2030)”, the health system, the Department of Agriculture and Rural Affairs and other departments have jointly carried out the “mask + gloves” special action, health education, sheep immunization and other measures; The special action of “cleaning and disinfection” has been carried out in key links such as sheep trading, slaughtering, and harmless treatment, which has effectively reduced the incidence of human brucellosis disease.

The majority of cases were males (71.01%), which is consistent with the results of the national and other provincial studies [42,43]. Human infection mainly associated with close contact between cattle and sheep [44,45]. This is because in rural areas, they are mainly engaged in high-risk jobs such as livestock immunization, slaughtering, trade, transport and fur processing. Despite recent efforts in health education highlighting the importance of protective equipment (e.g., gloves and masks), many rural residents remain at high risk due to low awareness and economic limitations [46]. Brucellosis occurred in all age groups, with a higher incidence among age group 35–74, especially those between 50–60 years, which was consistent with other studies [6].

Time cluster analysis indicated a clear seasonality in brucellosis, with most cases reported between February and September, peaking in June, aligning with other studies [23,36,47,48]. It may be related to a variety of factors, spring is the peak period of conservation, shearing, and immunization, during which people often perform high-risk tasks with bare hands [49]. In the summer, risks include handling newborn lambs, stillbirths, and amniotic fluid [50], as well as cleaning pens, all of which increase exposure to *Brucella* through aerosol or skin contact. Warmer temperatures and increased rainfall [51], also promote *Brucella* growth and reproduction [52]. Therefore, the Department of Health, Agriculture and Rural Affairs and other departments should vigorously carry out health education and public activities for key populations, promote the use of personal protective equipment, emphasize disinfection of the breeding environment and disposal of aborted lambs.

Cases have been reported in all 22 counties, consistent with the previous studies [21]. In Ningxia, sheep farming is primarily conducted through family-based, scattered backyard systems, where humans and animals often share the same courtyard, maintaining a high infection risk. Yanchit County, Tongxin County, Zhongning County, Xiji County and Yuanzhou District are all traditional agricultural and animal husbandry areas in Ningxia. The breeding of Yanchi Countys Tan sheep, Tongxin County and Zhongwei County’s beef cattle, and Xiji County and Yuanzhou District’s cattle and sheep are all local pillar industries. The number of breeding households in these areas is large, and the main farming methods are courtyard scattered farming and small-scale farming, with frequent contact between people and livestock [37]. They are also inhabited predominantly by ethnic minorities, where high-risk behaviors such as hand-feeding lambs and handling aborted fetuses/placentas are common [53]; among them, Yanchi County, which had the highest incidence, borders high-incidence regions such as Shaanxi, Gansu, and Inner Mongolia, and cross-border animal trade may introduce infected livestock. In contrast, the low incidence in Xingqing District (5.60/100,000) is mainly due its high urbanization, lower opportunities of human and animals contact and improved testing capabilities of medical institutions. It should be noted that counties such as Huinong, Qingtongxia, Pengyang, Haiyuan, and Lingwu, although not currently high-incidence areas, show fast annual growth, suggesting that their incidence rates may soon catch up or surpass current hotspots. These counties should be prioritized in future prevention and control efforts.

Spatial autocorrelation analysis showed that only the incidence rate of human brucellosis in Ningxia from 2022 to 2024 had spatial clustering, indicating that in most years (2010–2021) of the entire study period, the incidence rate of human brucellosis in each county unit was relatively random in spatial distribution, that is, mixed distribution of high and low counties. However, from 2022 to 2024, this pattern change, and there is a significant spatial clustering trend throughout the region, especially in 2023 when the clustering is most significant. Furthermore, the evolution of spatial clustering patterns offers critical insights. The presence of borderline significant spatial autocorrelation in several years prior to 2022, which then transitioned to strong and statistically significant clustering from 2022 to 2024, suggests that the spatial dependency of brucellosis in Ningxia was not abrupt but rather intensified over time. This shift is closely linked to a significant escalation of the epidemic driven by intensified cattle and sheep farming in Ningxia.

Local autocorrelation analysis found that the “high and high” clustering areas that appeared in 2010, 2011, and 2013, which were mainly in Zhongning County and Tongxin County, indicating persistent “hotspots”. However, this pattern was broken in the following decade. Four clustering areas were identified through spatiotemporal scanning analysis, including one most likely clustering area and three secondary clustering areas.The most likely cluster was centered on Jingyuan County with a radius of 271 km, encompassing areas from the Yellow River irrigation district in Ningxia to the southern mountains. This extensive area represents a widespread high-risk zone composed of multiple tighter clusters, rather than a single focus. The region is likely a hub for live animal transportation, with diseases potentially spread via highways to 12 surrounding counties, including the high-risk “high-high” clusters of Zhongning and Tongxin Counties. Consequently, prevention and control measures in these areas require strengthening.The radiation radius of the three secondary clustering areas gradually decreases, and the last secondary clustering area is only Helan County, which may have occupational exposure and present as a point source outbreak. Time dimension shows that the main clustering areas lasted for 5 years (2020–2024), synchronized with the large-scale transformation of animal husbandry in Ningxia, indicating that the transmission chain of brucellosis was not effectively blocked in the early stage, forming a cross regional transmission circle. The occurrence time lag of secondary clustering areas (2021–2023) may be related to the spillover of brucellosis outbreaks in the main clustering areas.

## Conclusion

This study shows that human brucellosis in Ningxia has shown a significant increasing trend, with a high epidemic intensity and clear seasonal characteristics. Middle aged and elderly individuals, as well as males, are the main affected population. Currently, the brucellosis epidemic is mainly concentrated in the Yellow River irrigation area to the southern mountainous areas of Ningxia, and there is a risk of spreading outward. Therefore, effective prevention and control of brucellosis requires coordinated efforts from multiple departments to minimize its harm ^to public health and the economy and society. Specifically, the Department of Agriculture and Rural Affairs and the market supervision department should focus on strengthening the control of infectious sources, especially via strictly supervising the cross-county transportation of cattle and sheep. The health department should carry out targeted health education for key populations. The study has three main limitation:

First, a key limitation stems from our reliance on national passive surveillance data. While this is the primary source for case information, it inherently and substantially underestimates the true disease burden by mild or misdiagnosed cases. This under-ascertainment may lead to an underestimation of the true incidence and could bias the identification of high-risk areas and risk factors if care-seeking behaviors vary geographically.Second, our spatial analysis was conducted at the county level, which introduces the potential for ecological fallacy. The risk factors inferred from these aggregated data represent average associations at a group level and may not accurately reflect risks at the individual level. For instance, an individual residing in a county classified as ‘high-risk’ may not necessarily possess the risk factors we identified. Therefore, causal inferences at the individual level should be drawn with caution.Third, this study focused solely on human case data. As discussed in the introduction, factors such as livestock prevalence, vaccination coverage in animal reservoirs, and environmental parameters are likely critical drivers of transmission. The absence of these data limits our ability to establish stronger causal inferences. Future studies that integrate human, animal, and environmental data are essential to unravel the complex transmission pathways of this disease.
